# Beta-interferons in multiple sclerosis: A single center experience in India

**DOI:** 10.4103/0972-2327.64624

**Published:** 2010

**Authors:** Salil Gupta, R. Varadarajulu, R. K. Ganjoo

**Affiliations:** Department of Neurology, Command Hospital Air Force, Bangalore, India

**Keywords:** Adverse events, beta-interferon, Indian patients, multiple sclerosis

## Abstract

**Background:**

Indian-Asian multiple sclerosis behaves somewhat differently from Western disease. It is not known if the response to β-interferon is also different.

**Aim:**

To demonstrate the decrease in relapses with β-interferon in Indian patients with multiple sclerosis.

**Patients and Methods:**

Patients with relapsing–remitting or secondary progressive multiple sclerosis with at least two relapses were started on β-interferon.

**Results:**

Sixteen patients were followed up for a period of 1–3 years. Fifteen had relapsing–remitting multiple sclerosis (MS). The mean number of relapses in these patients before interferons were started was 3.4. The mean yearly relapse rate was 1.3. The mean Kurtzke Expanded Disability Status Scale (EDSS) at the start of β-interferon therapy in relapsing–remitting MS was 1.7. Ten of these patients were on Avonex^®^ (interferon β1a) and six (including the patient with secondary progressive MS) were on Betaferon^®^ (interferon β1b). On follow-up, three patients (two on Avonex^®^ and one on Betaferon^®^ ) had relapses. The respective β-interferon being received by these patients was continued, with no further relapses. The remaining patients had no relapse or clinical or MRI progression after starting the drug. The side effect profile of the drug in these patients was favorable; although nearly all developed fever on the first day of the injection, only 50% of the patients continued to have fever after 3 months. Two patients developed psychiatric symptoms, requiring discontinuation of the drug.

**Conclusion:**

Our prospective follow-up study shows that β-interferons are safe and effective in Indian patients with relapsing–remitting or secondary progressive MS.

## Introduction

After the landmark trial in 1993[1] interferons assumed a central role in the management of multiple sclerosis (MS). Three different β-interferon (IFN-β) products are approved for the management of relapsing–remitting multiple sclerosis (RRMS) and, to a certain extent, secondary progressive multiple sclerosis (SPMS). The beneficial effects of β-interferon in MS are believed to be mainly related to its anti-inflammatory effects – which is based on reduced MHC class II expression and shift of cytokine production from Th1 to Th2 – as well as its direct effects in preserving the integrity of the blood–brain barrier.[2,3]

Previous studies have suggested that Indian and Asian patients with MS behave somewhat differently from their Western counterparts. They have greater visual impairment initially and more optic nerve and spinal involvement, among other differences.[4-12] It is not known if these clinical differences in the behavior of the disease translate into variation in the response rate to β-interferons. A PubMed search using the words 'multiple sclerosis,' 'India,' and 'beta-interferon' failed to reveal any published series on the use of β-interferons in Indian patients with MS.

Against this background, we did a prospective follow-up study of Indian patients with MS in whom β-interferons were indicated. The aims of the study were as follows:

- To demonstrate the decrease in relapses in these patients despite the previously documented variations in presentation when compared to Caucasian patients with MS
- To determine the adverse event profile of β-interferons

## Patients and Methods

### Patient population

This study was done at an armed forces tertiary care hospital located in south India and with a large dependent clientele. All patients were either serving or retired armed forces personnel or their dependents who were entitled to free treatment through the Armed Forces Medical Services.

### Inclusion criteria

Only those patients were included who had been diagnosed to have MS according to McDonald's criteria.[13] The diagnosis was based on a detailed history, clinical examination, gadolinium-enhanced MRI of brain and spine, visual evoked potentials (VEP), and presence of oligoclonal IgG bands in cerebrospinal fluid (CSF). The MRI criteria used were the same as those used in the diagnostic criteria, i.e., the criteria of Barkhof *et al*. as modified by Tintore *et al*.[14,15] Brainstem auditory evoked response (BAER) was also done in all patients who were included in the study.

Only patients who had at least two or more clinical attacks and were diagnosed to have RRMS or SPMS, as defined by an international survey,[16] were included in the study.

### Exclusion criteria

Those patients with primary progressive multiple sclerosis (PPMS) or with progressive relapsing MS were excluded from the study.

### Choice and dose of β-interferon

The choice of β-interferon was arbitrary. In this study either Avonex^®^ (interferon β1a) 30 µg intramuscularly once a week or Betaferon^®^ (interferon β1b) 250 µg subcutaneously every alternate day were used. We made no attempt to compare the relative efficacies of the two β-interferons.

### Baseline evaluation

Prior to starting β-interferon, each patient had their score on the Kurtzke Expanded Disability Status Scale (EDSS) recorded.[17]

### Follow-up evaluation

After starting interferon the patients were monitored monthly. During each visit a detailed history was taken and the findings on clinical examination were recorded with respect to progression of symptoms or appearance of new signs. EDSS and blood counts were done every 3 months. Gadolinium-enhanced MRI of brain and spine was done yearly.

### Clinical relapse

A clinical relapse was defined as the presence of a new symptom or sign lasting for at least 24 h and which was not associated with fever. If a patient had a relapse, the nature of the relapse was recorded, MRI was done, standard therapy for relapse was given, and the recovery was recorded.

### Clinical progression

Clinical progression in the absence of relapse was defined as change in EDSS by one point.

### Progression on MRI

Progression on MRI was defined as the appearance of a new gadolinium-enhancing lesion at a site different from that recorded earlier, appearance of a new T2-weighted lesion, or increase in the size of an old lesion.

### Comparison of follow-up MRI

The MRI done on follow-up was visually compared to the baseline MRI. The comparison was not blinded.

### Adverse event monitoring

With respect to the adverse events after β-interferon injection, the following data was recorded: appearance of fever, duration of fever, persistence of fever after 3 months, myalgia, headache, injection site pain, skin reactions, or psychiatric manifestation.

### Data recording and statistical analysis

All analysis was done after recording the data on a Microsoft Excel sheet. No statistical comparison was done among any groups.

## Results

Sixteen patients were diagnosed as MS and satisfied the inclusion criteria of the study. There were nine males. The mean age at onset of symptoms was 33.5 years (range 20–52 years). The first clinical feature at onset of disease and the clinical features encountered during the course of illness are shown in Table 1. Fifteen patients had RRMS and one had SPMS when β-interferon was initiated. VEP showed prolonged P100 latencies in 14 (87.5%) patients, while BAER was abnormal in 6 (37.5%). Oligoclonal band was detected in the cerebrospinal fluid (CSF) of 12/14 (85.7%) patients; two patients did not undergo CSF examination.

**Table 1:** Clinical features at onset and during the course of illness

Clinical feature	At onset (%)	During the course
Optic neuritis	9 (56.25)	10 (62.5)
Diplopia/bulbar symptoms	2 (12.5)	4 (25)
Motor involvement	4 (25)	13 (81.25)
Sensory involvement	3 (18.75)	12 (75)
Cerebellar symptoms	0	6 (37.5)
Bladder symptoms	0	9 (56.25)

### Baseline characteristics

RRMS: The mean duration of illness before starting β-interferon was 3.7 years (range: 0.5–13 years). The mean number of relapses before starting β-interferon was 3.4 (range: 2–6). The mean yearly relapse rate was 1.3 (range: 0.3–3). The mean EDSS score when β-interferon was started was 1.7 (range: 0–3).

### SPMS

The patient with SPMS had seven relapses initially. The last relapse was 2 years before starting β-interferon. After the last relapse he had shown progression of motor symptoms. At the time of initiating β-interferon the EDSS score was 6.

### Interferon distribution

Ten patients were initiated on Avonex^®^ and six on Betaferon^®^ in the doses mentioned earlier. The patient with SPMS received Betaferon^®^. The mean follow-up period was 2.25 years (range: 1–3.25 years).

### Relapses

Two patients on Avonex^®^ and one on Betaferon^®^ had relapses. All three relapses occurred in the first year after starting β-interferon and were documented with new MRI lesions. The recovery was complete after giving steroids. β-Interferon was continued in all three patients with no further relapses during a mean follow-up period of 1.5 years.

### Follow-up MRI

**Figure 1:**
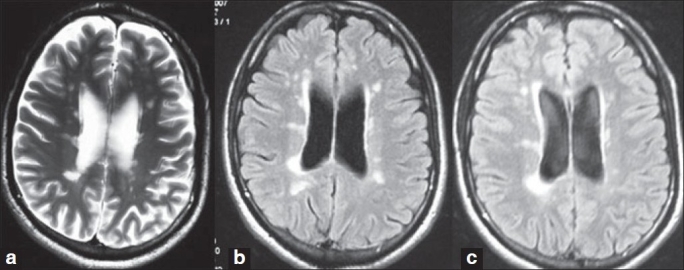
(a) Baseline (2006) MRI brain of one our patients with RRMS before starting IFN β. (b) Follow up (2007) MRI brain of patient showing no fresh lesion (c) Follow up (2008) MRI brain of patient showing no fresh lesion

**Figure 2:**
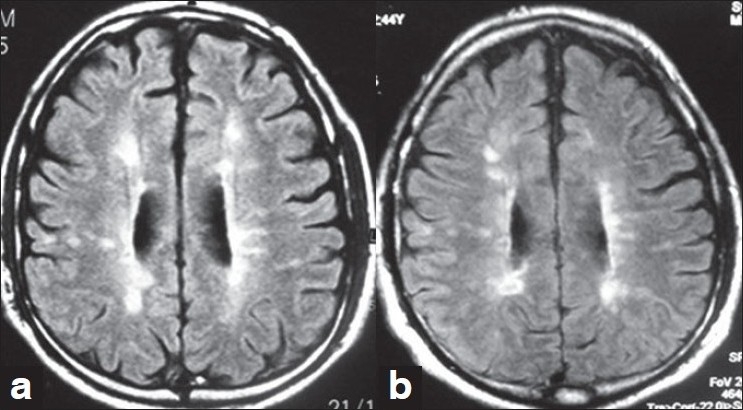
(a) Baseline MRI brain of patient with SPMS before starting IFN β. (b) Follow up MRI brain of patient with SPMS after starting IFN β

Visual comparison of follow-up MR images in patients with no relapses showed no fresh lesions. Figure 1a‐c show the serial MRIs of a patient. The patient with SPMS showed no further progression of EDSS score and no fresh lesions appeared on MRI [Figure 2a and b].

### Adverse events

β-Interferon had to be discontinued in two of our patients, both of whom were on Avonex^®^. The first patient was a known case of schizophrenia in remission and off all drugs. Seven years after discontinuing all drugs for schizophrenia, she had developed RRMS, with some residual deficit after each relapse. She was started on IFN β1a (Avonex^®^). Two months after starting the drug she had a relapse of the schizophrenic symptoms, with suicidal tendencies, and the drug had to be stopped. She was then started on glatiramer acetate; she has been taking this drug for the last 2 years and is relapse free. The second patient developed features of depression and suicidal ideas within 2 weeks of starting the β-interferon, and the drug had to be discontinued.

The other adverse events that occurred after initiating interferons are shown in Table 2. All patients developed fever after the first dose but, 3 months after the start of the injections, only 50% continued to have fever after each dose. The fever responded to a single dose of paracetamol in all cases. No patient had fever lasting for more than 24 h, the mean duration of the fever being 9 h. Myalgia and body aches occurred in nearly three-fourth of our patients after each injection. Headache was the other symptom reported. None of our patients reported injection site pain, skin changes, or necrosis.

**Table 2:** Adverse event profile

Adverse event	Number (%)
Fever after first dose	16 (100)
Persistent fever after 3 months	7/14 (50)
Duration of fever	9 (6–24) h
Headache	7 (43.75)
Myalgia and body ache	11 (68.75)
Injection site pain	0
Psychiatric symptoms	2
Leukopenia	0

## Discussion

Previous studies have brought out certain differences in the clinical profile of MS in India. One of the differences revealed in previous studies include older age at presentation,[8] though some Indian studies have also reported onset at slightly earlier age.[4,7,9,10] Male predominance has been documented in some Indian studies,[4,7] however there are other Indian studies that have documented female predominance.[8,9,11] Indian and Asian studies have documented visual impairment as a common initial symptom.[4,6,7,11,12] A few series have shown relatively little clinical involvement of the cerebellum[6,7,10,11]; however, this has been disputed since the advent of MRI, which has provided evidence of greater cerebellum involvement. The opticospinal variety of MS too has been reported more commonly in Indian patients.[7,11]

Most experts agree that the Indian-Asian patient with MS behaves a little differently from the Caucasian patient. Do these differences affect the response to β-interferon? This small case series attempted to answer this question. There are no documented case series of the use of β-interferons in MS in Indian patients.

This study has shown that after starting β-interferon all patients who could tolerate the drug had a significant reduction in the relapse rate. Most patients had no relapse at all during follow-up for a mean period of 2.25 years. There was no progression of clinical findings, as documented by a stable EDSS score, and no fresh lesions on MRI. The patient with SPMS too showed no clinical or radiological progression.

Overall, the drug was well tolerated. Most patients had minor side effects, as mentioned earlier.[17,18] β-Interferons should be avoided in patients with a history of major psychiatric symptoms.

Nearly 85% of our patients also had oligoclonal bands in the CSF. This figure is similar to those reported from the West in large series[19] as well as in some Indian series.[20] However, other series from India have reported lower figures,[9] implying a genetic basis. The high figure in our study could be due to the fact that all our patients had clinically active disease with a high relapse rate and a high lesion load on MRI.

Our study has shown that Indian patients with MS respond well to β-interferons. This is despite the view that Indian-Asian patients with MS have a slightly different clinical profile. Indian patients also tolerate the drug well. This study has implications for the management of Indian patients with MS and is the only study to date that has demonstrated the benefits of β-interferons in this population.
